# Effect of mindfulness-based stress reduction on stigma, coping styles, and quality of life in patients with permanent colorectal cancer stoma: A protocol for systematic review and meta-analysis

**DOI:** 10.1097/MD.0000000000028421

**Published:** 2022-01-07

**Authors:** Jing Li, En Yuan, Dan Zhu, Mei Chen, QingHua Luo

**Affiliations:** The National Hospital of Enshi Autonomous Prefecture, Enshi, Hubei Province, China.

**Keywords:** colorectal cancer, coping style, meta-analysis, mindfulness-based stress reduction, permanent colostomy, protocol, quality of life, stigma

## Abstract

**Background:**

: Colorectal cancer patients with permanent colostomy may suffer stigma, negative coping style, and low quality of life at varying degrees, which may be improved by the mindfulness-based stress reduction (MBSR). In recent years, MBSR has been used in the comprehensive treatment of colorectal cancer with permanent colostomy, hoping to bring a positive outcome. However, the practical application effect of MBSR has not been elucidated so far. Therefore, this study conducted a meta-analysis to evaluate the effects of MBSR on stigma, coping style, and quality of life in colorectal cancer patients with permanent colostomy, providing reliable evidence for clinical application.

**Methods:**

: Randomized controlled trials (RCTs) reporting MBSR on stigma, coping style, and quality of life in patients with permanent stoma of colorectal cancer published before December 2021 will be searched in online databases such as the PubMed, Web of Science, The Cochrane Library, Embase, China National Knowledge Infrastructure, Wanfang Database, China Biomedical Literature Database, and Chinese Scientific Journal Database. The quality of the literature will be evaluated using the risk of bias assessment tool in Revman 5.4. Meta-analysis will be performed using Revman 5.4 software.

**Results:**

: The Social Impact Scale (SIS), Simplified Coping Style Questionnaire (SCSQ), and quality of life scale will be used to evaluate the effects of MBSR on stigma, coping style, and quality of life in colorectal cancer patients with permanent colostomy.

**Conclusion:**

: This study will provide a reliable evidence-based basis for MBSR to reduce stigma, improve coping style, and improve quality of life for colorectal cancer patients with permanent colostomy.

**OSF REGISTRATION NUMBER:**

DOI 10.17605/OSF.IO/CD4PV.

## 1 Introduction

According to the 2018 Annual Report on Global Oncology, the prevalence of colorectal cancer ranks third following lung cancer and breast cancer.^[1]^ Low colorectal cancer is the main type of colorectal cancer.^[2,3]^ Due to the unsatisfactory effect of anal preservation treatment, abdominoperineal resection is mainly performed that requires a permanent colostomy.^[4,5]^ Great changes in the physicality, psychology, and social support result in the low self-esteem or shame of affected patients.^[6]^ It is reported that patients with permanent colostomy have varying degrees of stigma,^[7,8]^ which affects the coping style of patients and reduces the quality of life.^[9–11]^ Therefore, alleviating the stigma, improving coping styles, and improving quality of life have become the focus of current research.

Permanent colostomy not only changes the patient's original defecation pattern, but also affects the physicality, psychology, and social functions.^[5]^ Patients have a strong sense of shame, negative coping style, and impaired quality of life.^[5,12,13]^ At present, progressive focused interview, peer support therapy, and narrative therapy have been widely applied to improve stigma, coping style, and quality of life.^[14–16]^ However, the progressive focused interview method and peer support therapy have certain requirements on the practitioners, and the effects are not stable. MBSR comes from meditation, in which you look at yourself physically and mentally, change your mindset, and accept yourself.^[17]^ Some researchers have applied MBSR to colorectal cancer patients with permanent colostomy, and achieved good results in alleviating patients’ stigma, improving coping styles, and improving quality of life.^[18–20]^

The effect of MBSR on stigma, coping style, and quality of life of colorectal cancer patients with permanent colostomy is still unclear. This study aims to evaluate the impact of MBSR on stigma, coping style, and lifestyle of colorectal cancer patients with permanent colostomy through a meta-analysis, so as to provide evidence-based evidence for clinical development of MBSR.

## 2 Methods

### 2.1 Protocol register

This meta-analysis protocol is conducted based on the Preferred Reporting Items for Systematic Reviews and meta-analysis Protocols (PRISMA-P) statement guidelines. The protocol of the systematic review was registered on Open Science Framework, and the registration number is DOI 10.17605/OSF.IO/CD4PV.

### 2.2 Ethics

All data in this study were all from published literature, and therefore, there was no need to recruit patients and collect personal information. The approval of the ethics committee is not required.

### 2.3 Inclusion and exclusion of literature

Inclusion criteria included:

(1) Colorectal cancer patients with permanent colostomy who were able to complete the intervention and questionnaire;
(2) RCTs reporting the effects of MBSR on stigma, coping style, and quality of life of colorectal cancer patients with permanent colostomy;
(3) Intervention: Routine nursing performed in control group. MBSR, such as mindful meditation, body scan, mindful walking and mindful yoga, as well as routine nursing performed in observation group;
(4) Outcome measures: Social Impact Scale (SIS) score, Simplified Coping Style Questionnaire (SCSQ) score, and quality of life scale score.

Exclusion criteria included:

(1) Repeated publication of documents;
(2) Inability to obtain full documents;
(3) Incomplete data or inability to obtain original data.

### 2.4 Search strategy

RCTs reporting MBSR on stigma, coping style, and quality of life in patients with permanent stoma of colorectal cancer published before December 2021 will be searched in online databases like the P PubMed, Web of Science, The Cochrane Library, Embase, China National Knowledge Infrastructure, Wanfang Database, China Biomedical Literature Database, and Chinese Scientific Journal Database with the combination of MeSH terms and free words. References in eligible literatures will be manually reviewed. The searching strategy in the PubMed is shown in Table 1.

**Table 1:** Search strategy in PubMed database.

Number	Search terms
#1	Colorectal Neoplasms[MeSH]
#2	Colorectal Cancer[Title/Abstract]
#3	Colorectal Carcinoma[Title/Abstract]
#4	Colorectal Tumors[Title/Abstract]
#5	Neoplasms, Colorectal[Title/Abstract]
#6	Cancer, Colorectal[Title/Abstract]
#7	Cancers, Colorectal[Title/Abstract]
#8	Carcinoma, Colorectal[Title/Abstract]
#9	Carcinomas, Colorectal[Title/Abstract]
#10	Colorectal Cancers[Title/Abstract]
#11	Colorectal Carcinomas[Title/Abstract]
#12	Colorectal Neoplasm[Title/Abstract]
#13	Colorectal Tumor[Title/Abstract]
#14	Neoplasm, Colorectal[Title/Abstract]
#15	Tumor, Colorectal[Title/Abstract]
#16	Tumors, Colorectal[Title/Abstract]
#17	OR/1-16
#18	Enterostomy[MeSH]
#19	Enterostomies[Title/Abstract]
#20	Stoma[Title/Abstract]
#21	OR/18-20
#22	Mindfulness-based stress reduction [Title/Abstract]
#23	MBSR[Title/Abstract]
#24	Mindfulness [Title/Abstract]
#25	OR/22-24
#26	Randomized Controlled Trials as Topic[MeSH]
#27	Clinical Trials, Randomized[Title/Abstract]
#28	Controlled Clinical Trials, Randomized[Title/Abstract]
#29	Trials, Randomized Clinical[Title/Abstract]
#30	Random∗[Title/Abstract]
#31	OR/26-30
#32	#17 AND #21 AND #25 AND #31

### 2.5 Date screening and extraction

**Figure 1:**
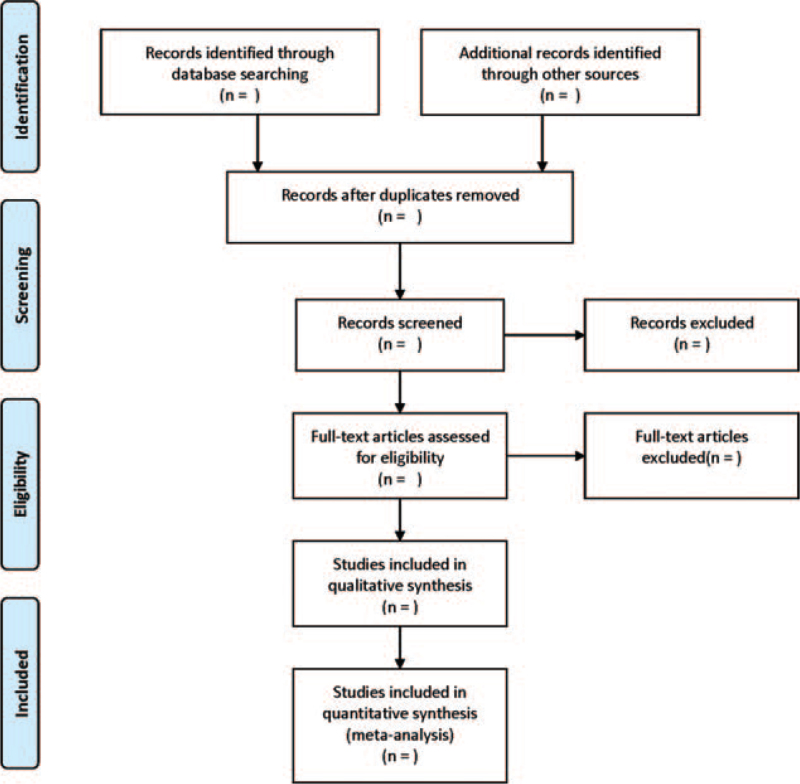
Flow diagram of study selection process.

Two reviewers will be independently responsible for literature searching. We will first review the title and abstract, followed by the full-text. Any disagreement will be solved by the third researcher through discussion. Two reviewers will be independently responsible for extracting the following data: first authors, country, date of publication, sample size, age, intervention details and intervention time of control group and observation group, outcome measures, etc. A preferred reporting items for systematic reviews and meta-analysis flow chart has been drawn to illustrate the study selection procedure (Fig. 1).

### 2.6 Evaluation of literature quality

The quality of the included studies will be evaluated according to the Cochrane manual of RCT risk assessment tool for bias^[21]^: The generation of random sequences; Allocation hidden; Whether participants adopt blind method; Whether the outcome evaluator adopts blind method; Integrity of outcome data; Selective reporting of research results; and Other biases. Literature quality will be assessed into grade A (low risk with 4 or more eligible items), B (moderate risk with 2 or 3 eligible items), and C (high risk with 0 or 1 eligible item).

## 3 Statistical analysis

### 3.1 Data analysis and processing

The RevMan 5.4 software will be used to meta-analysis. The standardized mean difference (SMD) of measurement indexes will be used as the effect index, and the 95% confidence intervals (95% CIs) will be calculated for interval estimation. The heterogeneity of included literatures will be assessed by the Chi-square test (α = 0.1), and quantified by *I*^2^ test. *P* ≥ .1 and/or *I*^2^ < 50% indicates no heterogeneity and a fixed-effect model will be used for pooled analysis; Otherwise, a random-effect model will be introduced.

### 3.2 Subgroup analysis

Subgroup analysis will be performed according to patients’ age, time of wearing colostomy, and time of intervention.

### 3.3 Sensitivity analysis

In order to ensure the stability of meta-analysis results, sensitivity analysis will be conducted by one-by-one elimination method.

### 3.4 Assessment of reporting Biases

When at least 10 studies are included in the present study, we will assess publication bias using funnel plots.

## 4 Discussion

Colorectal cancer patients with permanent colostomy have their vital organ (anus) been removed, and they require permanent colostomy.^[9]^ In the process of wearing the colostomy, it brings many troubles to patients, such as the change of defecation mode, body shape, odor, sound of excreta, and other people's strange eyes.^[11,22,23]^ To avoid these difficulties, patients with colostomy will try to avoid going out, reduce social activities, and even self-isolation.^[24]^ They have stigma because of the presence of colostomy.^[25]^ The resulting stigma not only aggravates the harm of the disease, but also changes the daily coping style and gradually decreases the quality of life, forming a vicious circle.^[5,26]^ However, the general intervention therapy has certain restrictions and unstable effect.

MBSR includes mindful meditation, body scan, mindful walking, mindful yoga, and mindful relaxation techniques,^[27]^ which improves the patient's level of mindfulness by establishing intention, focusing on momentary experiences, and developing an open and accepting attitude.^[28,29]^ Through a variety of mindfulness meditation, body awareness, and yoga to wake up the inner concentration, help individuals to decompress, and strengthen emotional management, the ability of physical and mental regulation can be improved to reduce the patient's sense of shame, change coping style, and improve the quality of life.^[30]^

However, our study also has some limitations:

(1) Most of the included literatures are published in Chinese, which may lead to publication bias.
(2) The number of included studies is small, and more high-quality literature should be included for further analysis in the future.
(3) The specific programs of MBSR may have a certain impact on heterogeneity due to the difference in the target.

## Author contributions

**Conceptualization:** QingHua Luo, Jing Li.

**Data curation:** Jing Li, En Yuan.

**Formal analysis:** Dan Zhu, En Yuan.

**Funding acquisition:** QingHua Luo.

**Investigation:** En Yuan.

**Methodology:** En Yuan, Mei Chen.

**Project administration:** QingHua Luo.

**Resources:** Dan Zhu.

**Software:** Dan Zhu.

**Supervision:** QingHua Luo.

**Validation:** Dan Zhu, Mei Chen, En Yuan.

**Visualization and software:** Mei Chen.

**Visualization:** Dan Zhu, Mei Chen.

**Writing – original draft:** Lei Wu, Hui Tan, QingHua Luo, Jing Li.

**Writing – review & editing:** Jing Li and QingHua Luo.
